# Supplementary material for: AXL receptor tyrosine kinase is required for T cell priming and antiviral immunity
Source: eLife. 2016 Jun 28;5:e12414. doi: 10.7554/eLife.12414 (PMC4924996; doi:10.7554/eLife.12414)
Supplement: Supplementary file 1. — F = forward, R = reverse. DOI: http://dx.doi.org/10.7554/eLife.12414.021 [file elife-12414-supp1.docx]

| Primers for RT-PCR | |
| --- | --- |
| *Gapdh* (mouse) | F: TCCCACTCTTCCACCTTCGA  R: AGTTGGGATAGGGCCTCTCTT |
| *PA* (A/PR8 polymerase acidic) | F: CGGTCCAAATTCCTGCTGAT  R: CATTGGGTTCCTTCCATCCA |

**Supplementary File 1.** Primer sequences for the indicated genes. F = forward, R = reverse
